# Exosomal RNF157 mRNA from prostate cancer cells contributes to M2 macrophage polarization through destabilizing HDAC1

**DOI:** 10.3389/fonc.2022.1021270

**Published:** 2022-10-03

**Authors:** Han Guan, Likai Mao, Jinfeng Wang, Sheng Wang, Shuai Yang, Hongliang Wu, Wenyan Sun, Zhijun Chen, Ming Chen

**Affiliations:** ^1^ Department of Urology, The First Affiliated Hospital of Bengbu Medical College, Bengbu, China; ^2^ Department of Urology, The Second Affiliated Hospital of Bengbu Medical College, Bengbu, China; ^3^ Department of Urology, Yancheng No. 3 People’s Hospital, Yancheng, China; ^4^ Department of Urology, Zhongda Hospital Affiliated to Southeast University, Nanjing, China

**Keywords:** exosomal RNF157 mRNA, prostate cancer, M2 macrophage polarization, HDAC1, ubiquitination

## Abstract

**Background:**

Exosomes have been identified to mediate the transmission of RNAs among different cells in tumor microenvironment, thus affecting the progression of different diseases. However, exosomal messenger RNAs (mRNAs) have been rarely explored. RNF157 mRNA has been found to be up-regulated in PCa patients’ exosomes, but the role of exosomal RNF157 mRNA in PCa development remains unclear.

**Methods:**

Online databases were utilized for predicting gene expression and binding correlation between different factors. RT-qPCR and western blot assays were respectively done to analyze RNA and protein expressions. Flow cytometry analysis was implemented to analyze M2 polarization.

**Results:**

RNF157 expression was high in PCa tissues and cells. M2 polarization of macrophages was enhanced after co-culture with PCa cells or with exosomes released by PCa cells. Upon RNF157 knockdown in PCa cells, the extracted exosomes could not lead to the facilitated M2 polarization. Mechanistically, RNF157 could bind to HDAC1 and contribute to HDAC1 ubiquitination, which led to HDAC1 degradation and resulting in promoting M2 polarization of macrophages. Animal experiments validated that exosomal RNF157 accelerated PCa tumor growth through facilitating macrophage M2 polarization.

**Conclusion:**

Exosome-mediated RNF157 mRNA from PCa cells results in M2 macrophage polarization *via* destabilizing HDAC1, consequently promoting PCa tumor progression.

## Introduction

Prostate cancer (PCa) is a heterogeneous disease that severely damages the health of men (1), since patients with PCa usually face problems such as urinary incontinence, bowel leakage and erection problems (2, 3). More importantly, due to the special location of the disease, the psychological health is also largely damaged (4). It causes nearly 360000 deaths across the world in 2018, ranking as the fifth leading cause of cancer-related death (5). Statistics show that the incidence of PCa is stable over the past years, but there is an increase of 4%-6% in that of advanced PCa since 2011 (6). Currently, active surveillance has been regarded as the prior treatment for patients with less-aggressive PCa, while surgery and radiation are effective for localized cases (7). A recent study has revealed that the intra-tumoral heterogeneity at the genomic, epigenetic and phenotypic levels in PCa has set obstacles for developing effective diagnostic or therapeutic targets (1). In this regard, it is imperative to find more efficient biomarkers to improve the early diagnosis and treatment.

There is a study reporting that exosomes are potentially used as diagnostic biomarkers or treatment targets (8). Additionally, existing evidence have suggested that exosomes can affect the progression of cancers *via* transmitting RNAs among different cells in the tumor microenvironment (9). More specifically, long non-coding RNA LNMAT2 carried by exosomes is transmitted from BCa cells to human lymphatic endothelial cells, thus facilitating the tumor lymphangiogenesis of bladder cancer (10). Moreover, microRNA-205 (miR-205) packaged by exosomes from ovarian cancer cells has also been uncovered to accelerate angiogenesis of ovarian cancer (11). Aside from the widely discovered role of exosomal non-coding RNAs, the role of protein-coding messenger RNAs (mRNAs) is largely unexplored. Previously, RNF157 mRNA has been reported to be up-regulated in both exosomes and tumors of PCa (12). RNF157 is an E3 ubiquitin ligase which has been verified to affect cell cycle through acting as the downstream effector of PI3K and MAPK pathways (13). Nevertheless, the role of the exosome-mediated RNF157 in PCa is still elusive. In this study, we aimed to investigate the role of exosomal RNF157 mRNA in PCa.

It is well-known that exosomes are the main mediator of communications in tumor microenvironment (TME) (14). Except cancer cells, tumor-associated macrophages (TAMs) are one of the main components in TME (15). Macrophages are versatile immunocytes that exert various influences on biological processes in cancer cells, including the modulation of tissue homeostasis, defending against pathogens and helping wound healing (16). Nowadays, accumulating reports have unveiled the regulation of tumor-derived exosomes on macrophage polarization (17).

In brief, this study tried to figure out whether and how exosomal RNF157 mRNA from PCa cells could affect the polarization of macrophages. Our findings are expected to contribute to a more comprehensive understanding of PCa microenvironment.

## Materials and methods

### Cell culture

The human normal prostatic epithelial cell line (RWPE-1), procured from American Type Culture Collection (ATCC; Manassas, VA, USA), was maintained in Keratinocyte Serum-Free Growth Medium supplemented with 5 ng/mL human recombinant epidermal growth factor (EGF; Invitrogen, Carlsbad, CA, USA) and 25 mg/mL bovine pituitary extract (BPE; 10744-019, Gibco, Rockville, MD, USA). PCa cell lines (PC-3, LNCaP, DU145 and VCap) and human acute monocytic leukemia cells (THP-1), also acquired from ATCC, were maintained in RPMI 1640 medium (A4192301, Gibco) containing 10% fetal bovine serum (FBS; 100 units/ml; 16140071, Thermo Fisher Scientific, Rockford, IL, USA) and Penicillin-Streptomycin solution (100 µg/ml; 15070-063, Gibco). DMEM medium (A4192101, Gibco) with 10% FBS and 2mM glutamine was applied for culturing 293T obtained from ATCC too. All cultivation condition was controlled at 37°C with 5% CO_2_. To induce the transformation of THP-1 into macrophages, 185 ng/ml PMA (S-016-5, Gibco) was used to treat the cells for 6 h. Later, the morphology of THP-1 and PMA-induced M0 macrophages was observed under a light microscope (DMi1, Leica, Wetzlar, Germany).

### Cell transfection

Invitrogen provided all the plasmids for transfection. Short hairpin RNAs (shRNAs) were synthesized for knocking down of respective genes, including sh-RNF157-1/2/3 and sh-HDAC1-1/2/3. The RNF157 overexpression plasmid (pcDNA3.1-RNF157) and empty vector (pcDNA3.1) were also synthesized. Cells were transfected using Lipofectamine 2000 (11668019, Invitrogen) transfection reagent according to the instructions.

### Exosome isolation and PKH26 labeling

The procedures were in line with the description in a previous study (18). For exosome isolation, the plasma and culture medium were collected and centrifuged at 3000 × g for 15 min to remove cells and cellular debris. Exosomes were isolated using the Exoquick exosome precipitation solution (AM9480, Invitrogen).

Then, for exosome labeling, exosomes were suspended in PBS (SH30256.01B, Hyclone, Logan, UT, USA) containing PKH26 (V-22889, Invitrogen). The mixture was incubated for 4 min at room temperature. Then, this procedure was terminated after adding 2 ml of 0.5% bovine serum albumin (BSA; 23208, Thermo Fisher Scientific). The exosomes were harvested and were suspended in the basal medium, and 250 μl was added to the sub-confluent layer of cells. After incubation for 3 h at 37°C, cells were washed and fixed at room temperature. To stain the nuclei, DAPI (D9542, Sigma-Aldrich, St. Louis, MO, USA) was added for 10 min, and the stained cells were observed with a fluorescence microscope (DMI8, Leica).

### Exosome observation and identification

Exosomes suspended in 100 μl of PBS were then fixed with 5% glutaraldehyde (G6257, Merck, Darmstadt, Germany) and maintained at 4°C until transmission electron microscopy (TEM) analysis. According to the TEM sample preparation procedure, a drop of exosome sample was placed on a carbon-coated copper grid and immersed it in 2% phosphotungstic acid solution (pH 7.0; P4006, Merck) for 30s. The preparations were observed with a transmission electron microscope (G1403, Merck). Exosome quantification was determined *via* Nanosight Nanoparticle Tracking Analysis (NTA) as previously described (19). Briefly, PBS was utilized for resuspending the extracted exosomes. Subsequently, a syrine filter (SLHN033, Merck) was applied for filtering the exosomes. Ultimately, the size distribution of the diluted exosome samples was analyzed with a NanoSight NS300 instrument (Malvern PANalytical, Xuhui District, Shanghai, China). For determination of the exosome marker proteins (CD63 and TSG101) and calnexin (as negative control), the proteins were first extracted using extraction buffer with a protease inhibitor cocktail (P8340, Merck). The extracted proteins were then subjected to western blot analysis.

### Reverse transcription quantitative real-time polymerase chain reaction (RT-qPCR)

The total RNA was isolated from tissues or cell lines using TRIzol reagent (15596026, Invitrogen), while exosomal RNA was extracted from plasma and culture medium using the exoRNeasy Midi Kit (77144, QIAGEN, New York, NASDAQ) based on user’s protocol. The cDNA was synthesized using a high capacity cDNA reverse transcription kit (4368813, Applied Biosystems, Foster city, CA, USA). Quantitative real-time PCR (qRT-PCR) was conducted with SYBR Green Kit (F410L, Thermo Fisher Scientific). GAPDH was used to normalize mRNA expression levels. The expression of genes was counted by 2^-ΔΔCT^.

### Western blot

Proteins were prepared with a detergent buffer (R0278, Sigma-Aldrich), and the protein concentration was determined using the Pierce™ BCA Protein Assay Kit (20164, Thermo Fisher Scientific)y. Equal amounts (60 μg) of protein samples were separated by a 12% gel using sodium dodecyl sulfate-polyacrylamide gel electrophoresis (SDS-PAGE; 1610174, Bio-Rad Laboratories, Shanghai, China) and transferred onto polyvinylidene fluoride (PVDF; IPVH00010, Millipore, Bedford, MA, USA) membranes. After block by skim milk (37587, Thermo Fisher Scientific) for 1 h, the samples were then incubated with respective primary antibodies including anti-RNF157 (WH0114804M1), anti-PLRG1 (SAB2500805), anti-SMU1 (SAB1407636), anti-CHD1 (ZRB1692) and anti-PSMD8 (SAB1406325) provided by Sigma-Aldrich, and anti-β-actin (PA1-46296), anti-CD63 (MA1-19281), anti-TSG101 (1062BD), anti-calnexin (PA5-34665), anti-HDAC1 (49-1025), anti-RAN (48-2300), anti-EMD (701503) and anti-FXR2 (MA1-5773) provided by Invitrogen overnight at 4°C, followed by cultivation with secondary antibodies marked by horseradish peroxidase (HRP) (32260, Invitrogen) at room temperature for 2 h. Eventually, enhanced chemiluminescence (ECL) detection system (32209, Thermo Fisher Scientific) was applied for gray-scale value analysis.

### Chlorhexidine (CHX) and MG132 treatment

To inhibit protein synthesis in M0 macrophages, CHX (10 nM; HY-112951, MedChemExpress, New Jersey, USA) was added for 0/6/12-hour treatment. MG132 (100 nM; HY-13259C, MedChemExpress), a proteasome inhibitor, was utilized to rescue protein from degradation. Total protein extracted from CHX/MG132-treated M0 cells transfected with pcDNA3.1 or pcDNA3.1-RNF157 was subjected to western blot analysis. Additionally, HDAC1 mRNA level in cells after CHX treatment was also measured *via* RT-qPCR.

### Flow cytometry

For macrophage surface markers determination by flow cytometry, the harvested cells were fixed with paraformaldehyde (158127, Merck) overnight at 4°C. After overnight incubation, the cells were washed and then resuspended in flow cytometry buffer (1 × PBS buffer containing 1% FSA; 00-4222-57; Invitrogen). Then, Anti-CD163 (ab182422, Abcam, Cambridge, MA, USA) was used to stain the cells for 30 min at 20°C. After staining, the cells were washed, resuspended, and analyzed using a FACSCalibur flow cytometry (BD Biosciences, USA) according to the manufacturer’s instructions.

### Co-immunoprecipitation (CoIP)

This assay was carried out as previously described (20). For preparation, HEK193T and M0 cells were washed in PBS for three times. Then, the cells were lysed in lysis buffer containing complete protease inhibitors and centrifuged at 14,000 × g for 20 min at 4°C. The supernatant were incubated with Anti-RNF157 (ab185099, Abcam) or Anti-IgG (sb218427, Abcam) antibodies and protein A/G beads (HY-K0202, MedChemExpress) for 4 hours at 4°C to obtain the immunoprecipitated. The immunocomplexes were subjected to western blot assay after washing the beads.

### Immunoprecipitation and western blot (IP-WB)

This assay was conducted following previous description (21). Above all, IP lysis buffer (20-188, Millipore) was utilized for gathering MG132-treated/-untreated M0 cells in advance. To collect the supernatants, the cell lysates were subjected to 20-minute centrifugation at 16,000 × g. Subsequently, the collected supernatants were pre-cleared with protein A/G beads, followed by 2-hour incubation with primary antibody of Ubiquitin (ab140601, Abcam). Then, the immunoprecipitated protein complexes were collected and eluted, followed by transferring to SDS-PAGE gel. Eventually, the eluted samples and input fractions were subjected to western blot analysis.

### Animal experiment

The PC-3 cell lines (1 × 10^7^cells in 0.1 ml of PBS) were stably transfected with sh-NC or sh-RNF157-1 (PC-3/sh-NC and PC-3/sh-RNF157-1). The male nude mice were obtained from Slac Laboratories (Shanghai, China) and randomly divided into 4 groups (4 in each group). Then, PC-3/sh-NC or PC-3/sh-RNF157-1 were injected subcutaneously into the right flank of mice (n=4 for PC3/sh-NC and n=12 for PC3/sh-RNF157-1). After inoculation for 5 days, 4 of 12 mice with PC3/sh-RNF157-1 were treated with PBS, while another 4 of the 12 mice were treated with exosomes from PC3 cells (PC3/Exos). In other word, four groups of mice were involved in this work: PC3/sh-NC, PC3/sh-RNF157-1, PC3/sh-RNF157-1+PBS, and PC3/sh-RNF157-1+PC3/Exos. Tumor growth was examined every 3 days since day 7 by calculating the tumor volume as the formula: (length [mm] × width [mm]^2^)/2). After 4 weeks, all mice were sacrificed, and tumor size and weight were measured. The tumor volume was calculated as the formula: (length [mm] × width [mm]^2^)/2). The animal studies were approved by the Ethics Committee of the First Affiliated Hospital of Bengbu Medical College.

### Immunohistochemistry (IHC)

The tissue samples obtained from the excised xenegraft tumors from the animal experiment were fixed with 4% paraformaldehyde. Subsequent to dehydration and embedding in paraffin, the samples were sectioned into slices, followed by washing with 3% H_2_O_2_ deionized water (611006000009, Thermo Fisher Scientific). Afterward, these sections were blocked and incubated with primary antibodies of Ki67 (701198, Invitrogen) overnight at 4 °C. Following PBS washing, the corresponding secondary antibody (SA5-10262, Invitrogen) was added for one-hour incubation at room temperature. For the staining of nuclei, 3,3’-diaminobenzidine (DAB; 34065, Thermo Fisher Scientific) was used to produce a brown precipitate. A fluorescence microscope was applied for observing the stained cells.

### Statistical analyses

Each experiment in our study was performed in triplicate. Data were represented as means ± standard deviation (SD). GraphPad Prism 5 software (GraphPad Software, San Diego, CA) was applied for statistical analysis. Student’s t test was applied to compare statistical differences between two groups. In terms of data differences among three or more groups, one-way analysis of variance (ANOVA) was utilized. P < 0.05 was considered as statistically significant.

## Results

### RNF157 presents a high expression in PCa

**Figure 1:**
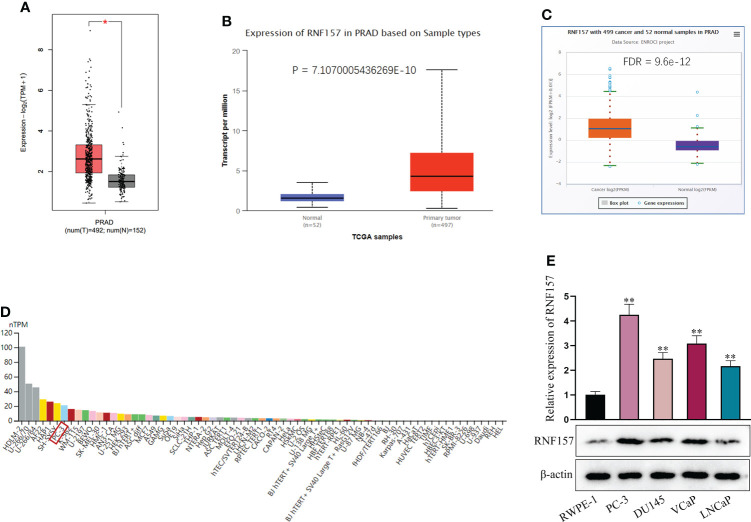
RNF157 was overexpressed in PCa. **(A–C)** GEPIA, UALCAN and ENCORI were applied to search for expression of RNF157 in PRAD tissues. **(D)** RNF157 expression in different cell lines, including PCa cell line (PC-3), was predicted on THE HUMAN PROTEIN ATLAS. **(E)** RNF157 expression in different PCa cell lines and in RWPE-1 cell line was analyzed *via* RT-qPCR and western blot. ^*^P < 0.05, ^**^P < 0.01.

In this study, we first obtained the expression pattern of RNF157 in PCa from multiple online databases, including GEPIA (http://gepia.cancer-pku.cn/), UALCAN (http://ualcan.path.uab.edu/index.html) and ENCORI (http://starbase.sysu.edu.cn/index.php). As displayed in **Figures 1A–C**, RNF157 expression was found to be aberrantly high in prostate adenocarcinoma (PRAD) tissues in comparison with normal tissues. Furthermore, data from THE HUMAN PROTEIN ATLAS (https://www.proteinatlas.org/) indicated that RNF157 expression in PC3 (a kind of PCa cell line) was higher than that in most of other cancer and non-cancer cell lines (**Figure 1D**). We also confirmed the up-regulation of RNF157 in PCa cell lines including PC-3, DU145, VCaP and LNCaP, relative to human prostatic epithelial cell line RWPE-1. Noteworthily, RNF157 displayed the most high expression level in PC-3 cells, among the PCa cell lines used in this study (**Figure 1E**). Thus, PC-3 was decided as the major research object involved in the subsequent assays. To conclude, RNF157 is overexpressed in PCa tissues and cells.

### Exosome mediates the transmission of RNF157 mRNA from PCa cells to macrophages

Currently, TAMs have been identified as the major element in TME, our emphasis was on the communication between PCa cells and macrophages. Accordingly, we treated THP-1 cells with PMA to induce macrophages. It manifested that the morphology of THP-1 was transferred into that of macrophages by PMA treatment; and RT-qPCR detection also unveiled the up-regulation of CD68 (the macrophage marker) in THP-1 cells under PMA treatment (**Figure S1A**). Results indicated that THP-1 cells treated with PMA (also mentioned as M0 cells subsequently) were successfully transformed into macrophages. Then, we co-cultured the macrophages with PC-3 cells and detected the percentage of CD163 (M2 polarization marker)-positive macrophages through flow cytometry analysis. It was found that M2 polarization of macrophages was facilitated after co-culturing with PC-3 cells, but such trend could be reversed under additional GW4869 (an inhibitor of exosome generation) treatment (**Figure S1B**). Moreover, RT-qPCR result showed the expression of ARG1 and IL-10 (M2 markers) was enhanced after M0 cells co-cultured with PC-3, but GW4869 abrogated the promoting effect of the PC-3 cell co-culture on M2 polarization of macrophages (**Figure S1C**). Above observations jointly evidenced that the influence of PC-3 cell co-culture on M2 polarization of macrophages was mediated by exosomes.

**Figure 2:**
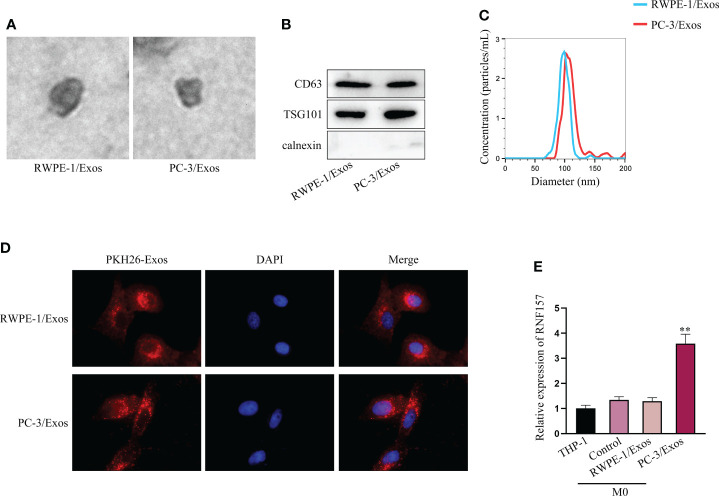
RNF157 mRNA from PCa cells could be transmitted to macrophages by exosomes. **(A)** Exosome morphology was observed through TEM. **(B)** The levels of exosomal markers (CD63 and TSG101) were tested through western blot. **(C)** The diameters of extracted exosomes were analyzed through NTA. **(D)** PKH26 was labeled to tract the exosomes and IF detected the labeled exosomes absorbed by macrophages. **(E)** RT-qPCR measured RNF157 mRNA level in M0 cells before and after co-culturing with exosomes from different cells. ^**^P < 0.01.

Thereafter, we isolated exosomes from RWPE-1 and PC-3 cells (labeled as RWPE-1/Exos and PC-3/Exos, respectively), and observed the morphology of exosomes under TEM (**Figure 2A**). Western blot indicated that CD63 and TSG101 (exosome markers) were expressed, but calnexin was not (endoplasmic reticulum marker) (**Figure 2B**). Based on NTA, the diameter of most exosomes was 50-110 nm, without aberrant difference between the size of RWPE-1/Exos and PC-3/Exos (**Figure 2C**). Next, we co-cultured M0 macrophages with PKH26-labeled exosomes extracted from PC-3 cells and observed that the PKH26-labeled exosomes could be absorbed in M0 cells (**Figure 2D**). Moreover, RNF157 mRNA level was noticed to be specially elevated in M0 cells after co-culture with PC-3/Exos, but that showed no change after co-culture with RWPE-1/Exos (**Figure 2E**). In sum, RNF157 mRNA can be transferred by exosomes from PCa cells to macrophages.

### Exosomes from PCa cells facilitate M2 polarization of macrophages *via* transmitting RNF157 mRNA

**Figure 3:**
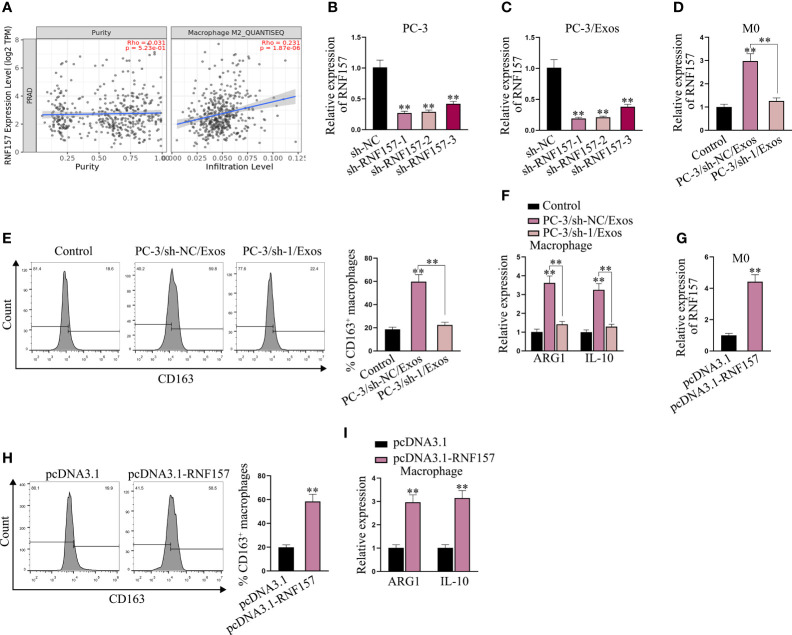
Exosomal RNF157 mRNA from PCa cells could induce the M2 polarization of macrophages. **(A)** TIMER 2.0 predicted the correlation between RNF157 expression in PRAD and M2 macrophage infiltration. **(B)** RNF157 was knocked down in PC-3 cells by transfection of sh-RNF157. **(C)** RNF157 mRNA level in exosomes from RNF157-depleted PC-3 cells was detected *via* RT-qPCR. **(D)** RNF157 mRNA level in macrophages after co-culture with exosomes from sh-NC or sh-RNF157 transfected PC-3 cells was tested through RT-qPCR. **(E)** Flow cytometry analyzed the percent of CD163-positive macrophages. **(F)** RT-qPCR analyzed ARG1 and IL-10 levels in macrophage after co-culture with different exosomes. **(G)** RT-qPCR the RNF157 level in macrophages after transfection of pcDNA3.1-RNF157. **(H, I)** Flow cytometry analysis of CD163 positivity and RT-qPCR analysis of ARG1 and IL-10 levels assessed M2 polarization rate before and after RNF157 augment. ^**^P < 0.01.

Next, we aimed to elucidate the influence of exosomal RNF157 mRNA on macrophage M2 polarization. From the prediction on TIMER 2.0 (http://timer.cistrome.org/), we noticed that RNF157 expression in PRAD was positively correlated with M2 macrophage infiltration (**Figure 3A**). It hinted that RNF157 might affect the M2 polarization in PCa. To investigate the influence of RNF157 on M2 polarization, a series of experiments were designed and carried out. Above all, we confirmed the high efficacy of RNF157 knockdown in PC-3 cells (**Figure 3B**) and further observed that the level of RNF157 mRNA in exosomes secreted by RNF157-depleted PC-3 cells was down-regulated accordingly (**Figure 3C**). Moreover, RNF157 mRNA in macrophages co-cultured with exosomes derived from RNF157-depleted PC-3 cells (PC-3/sh-1/Exos) also showed a decreased trend, comparing with that co-cultured with exosomes derived from control PC-3 cells (PC-3/sh-NC/Exos) (**Figure 3D**). Then, it was worth noting that M2 polarization of macrophages was promoted in PC-3/sh-NC/Exos group, but such variation was hardly seen in PC-3/sh-1/Exos group (**Figure 3E**). The detection of M2 polarization markers ARG1 and IL-10 further evidenced that co-culture with exosomes from sh-NC-transfected PC-3 cells could enhance M2 polarization, while exosomes from sh-RNF157-transfected PC-3 cells could not result in such change (**Figure 3F**). These findings suggested it was RNF157 mRNA transmitted by exosomes from PC-3 cells that induced the M2 polarization of macrophages. Furthermore, we overexpressed RNF157 in M0 cells (**Figure 3G**). As illustrated in **Figure 3H**, RNF157 augmentation could facilitate M2 polarization of macrophages, which was further evidenced by the increased ARG1 and IL-10 expressions in RNF157-overexpressed macrophages (**Figure 3I**). To sum up, RNF157 mRNA transferred by PCa-derived exosomes is able to induce M2 polarization of macrophages.

### RNF157 facilitates HDAC1 ubiquitination and degradation in macrophages

**Figure 4:**
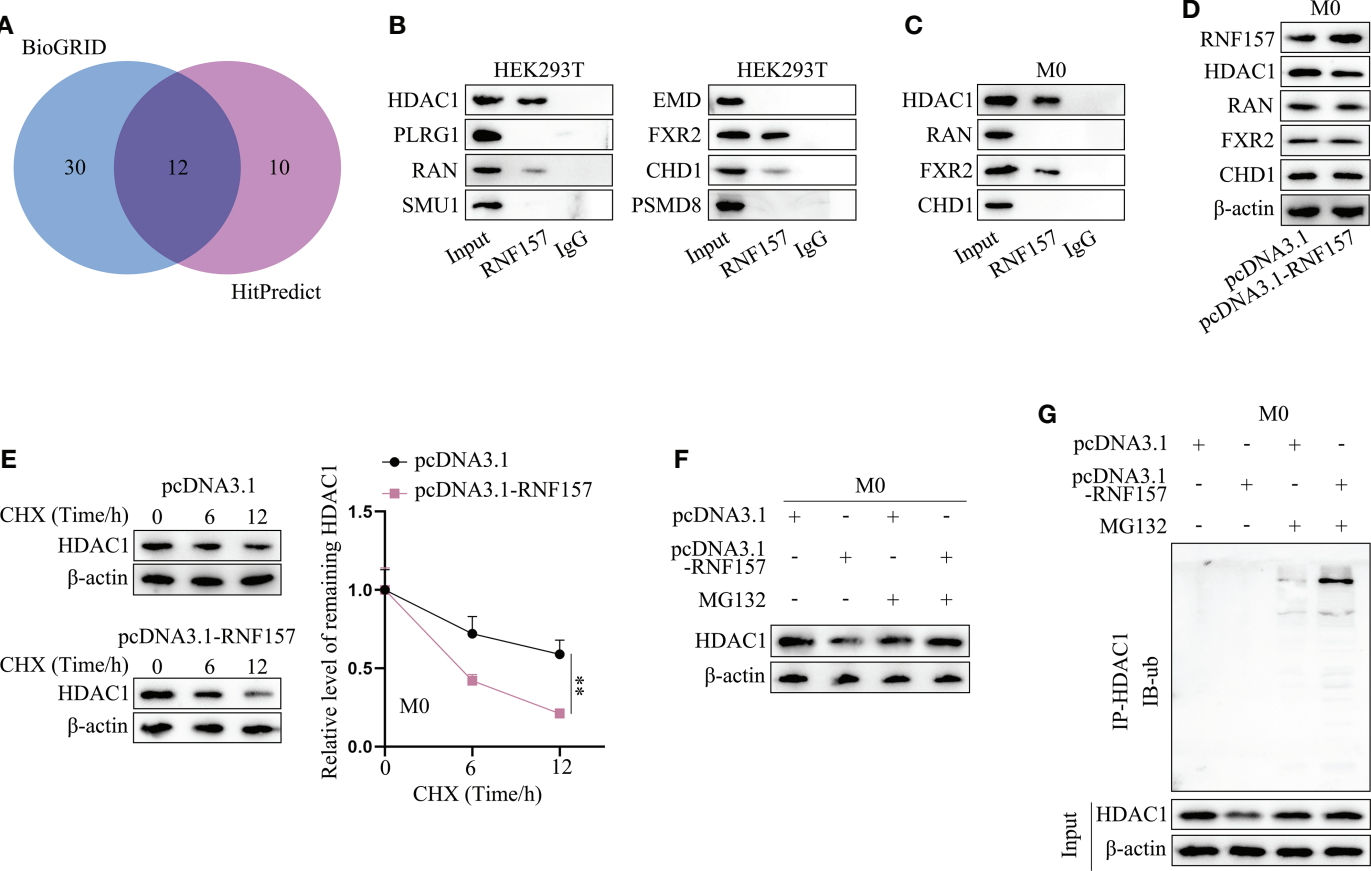
RNF157 facilitated HDAC1 ubiquitination and degradation in macrophages. **(A)** Proteins with binding potential to RNF157 were predicted on BioGRID and HitPredict. **(B)** CoIP analyzed the cohesion between RNF157 protein and the candidates in HEK293T. **(C)** CoIP detected the binding between candidates and RNF157 proteins in macrophages. **(D)** The expression of candidate proteins was analyzed by western blot in macrophages with or without RNF157 elevation. **(E)** The level of HDAC1 in macrophages at different time points after CHX treatment was measured through western blot. **(F)** The protein levels of HDAC1 were tested by western blot in macrophages under MG132 treatment when RNF157 was up-regulated or not. **(G)** Ubiquitination of HDAC1 in macrophages with or without RNF157 overexpression was analyzed *via* IP-WB. ^**^P < 0.01.

Our next target was to figure out the specific mechanism through which RNF157 induced M2 polarization of macrophages. Published literature has pointed out that the E3 ubiquitin ligase RNF157 could bind to target proteins and induce their ubiquitination (22). On this basis, we wondered whether there existed downstream proteins targeted and regulated by RNF157 in macrophages. Therefore, the binding protein of RNF157 was predicted through BioGRID (https://thebiogrid.org/) (Screening condition: High throughput) and HitPredict (http://www.hitpredict.org/) (Screening condition: High confidence), and 12 proteins with high possibility were selected (HDAC1, PLRG1, RAN, MEGF8, SMU1, EMD, FXR2, TECR, ATRN, MAGT1, CHD1 and PSMD8) (**Figure 4A**). Afterwards, we predicted the subcellular location of RNF157 as well as the candidate proteins through Hum-mPLoc 2.0 database (http://www.csbio.sjtu.edu.cn/bioinf/hum-multi-2/) to screen possible protein interactors with the same subcellular localization. As RNF157 demonstrated cytoplasmic and nuclear distribution, eight candidate proteins, including HDAC1, RAN, FXR2 and PSMD8 (existing in both cytoplasm and nucleus), together with PLRG1, SMU1, EMD and CHD1 (locating mainly in nucleus) owned the possibility to interact with RNF157 protein (**Figure S2**). Subsequently, CoIP was conducted in HEK293T cells to detect the cohesion between RNF157 and the 8 candidates. As a result, the binding potential of HDAC1/FXR2 to RNF157 was detected to be strong, while that of RAN and CHD1 was relatively weak, and the rest 4 candidates were excluded as they had no binding affinity to RNF157 (**Figure 4B**). Additionally, HDAC1 and FXR2 were observed to be able to combine with RNF157 in macrophages, and the binding affinity between HDAC1 and RNF157 was much stronger than the other two candidate interactors (**Figure 4C**). To analyze the regulatory influence of RNF157 on the downstream protein interactors, we overexpressed RNF157 in M0 cells and noticed an obvious decline of HDAC1 protein level (**Figure 4D**). Based on the abovementioned findings, only HDAC1 was involved in the follow-up investigation as the downstream interactor of RNF157 in M0 cells. Then, we used CHX (23) to inhibit the protein synthesis in cells, and analyzed the impact of RNF157 overexpression on the degradation of HDAC1 protein. It was illustrated that RNF157 up-regulation resulted in promoted HDAC1 protein degradation (**Figure 4E**). Then, MG132, the proteasome inhibitor (24), was utilized to treat M0 cells. It was corroborated that RNF157-overexpression-induced decline in HDAC1 protein level did not occur on account of MG132 treatment (**Figure 4F**). From IP-WB, we also saw that RNF157 enhanced the ubiquitination of HDAC1 (**Figure 4G**). In conclusion, RNF157 facilitates the ubiquitination and degradation of HDAC1 to negatively modulate HDAC1 expression in macrophages.

### Exosomal RNF157 mRNA suppresses HDAC1 expression to induce M2 polarization of macrophages

**Figure 5:**
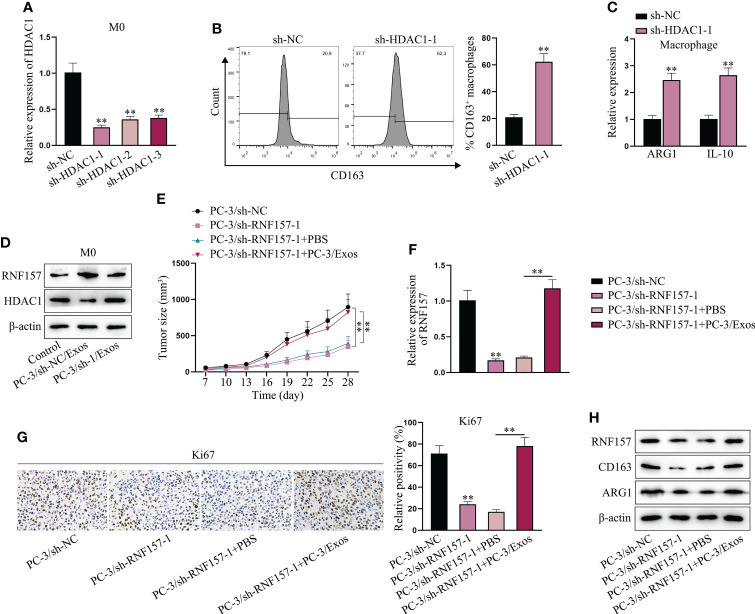
Exosomal RNF157 mRNA suppressed HDAC1 expression to induce the M2 polarization of macrophages. **(A)** RT-qPCR detected the HDAC1 expression in macrophages after transfection of sh-HDAC1-1/2/3. **(B, C)** Flow cytometry and RT-qPCR analyzed M2 polarization before and after HDAC1 depletion. **(D)** The protein levels of RNF157 and HDAC1 were analyzed by western blot in macrophages after co-culturing with PC-3/sh-NC/Exos or PC-3/sh-RNF157-1/Exos. **(E)** The growth curves of tumors were analyzed. **(F)** The expression of RNF157 mRNA in tumors was analyzed *via* RT-qPCR. **(G)** IHC detected Ki67 level in tumor tissues from different groups. **(H)** The protein levels of RNF157 and M2 polarization markers (CD163 and ARG1) were analyzed in tumor tissues from different groups. ^**^P < 0.01.

Next, experiments were designed to explore whether exosomal RNF157 facilitated M2 polarization of macrophages *via* suppressing HDAC1 expression. The inhibition efficiency of sh-HDAC1 was validated in advance (**Figure 5A**). Then, we found that CD163-positive cell percentage was increased when HDAC1 was depleted (**Figure 5B**), implying M2 polarization was promoted after HDAC1 depletion. Such conclusion was further supported, asARG1 and IL-10 expression was observed to be increased in HDAC1-depleted macrophages (**Figure 5C**). Moreover, opposite to the ascending trend of RNF157 protein level, HDAC1 protein level was observed to be decreased in macrophages treated with PC-3/sh-NC/Exos, while that in PC-3/sh-1/Exos group was similar to the control group (**Figure 5D**). Through *in vivo* study, we saw that RNF157 down-regulation in PC-3 cells could inhibit xenograft tumor growth, but PC-3-derived exosomes could abrogate the former suppressive impact (**Figure 5E**). RT-qPCR results confirmed that the down-regulated RNF157 expression in the tumor tissues obtained from PC-3/sh-RNF157-1 group could be rescued after additional injection of PC-3 exosomes (**Figure 5F**). Moreover, IHC assay revealed that Ki67 expression in tumor tissues was reduced by RNF157 interference, but rescued after additional treatment of PC-3-secreted exosomes (**Figure 5G**). Consistently, we also verified that the trend of CD163 and ARG1 levels was the same with that of RNF157 level in the excised tumors (**Figure 5H**), revealing the positive correlation of RNF157 expression with M2 macrophage infiltration in PCa tumors. To sum up, RNF157 mRNA transmitted by PCa-cell-derived exosomes contributes to M2 polarization of TAMs to aggravate PCa progression.

## Discussion

Extracellular vesicles have three major subtypes, including exosomes, microvesicles and apoptotic vesicles (25). Exosomes, ranging from 30-100 nm in size, are secreted by all kinds of cells and can be detected in the body fluids (26). All extracellular vesicles including exosomes communicate with other cells to modulate the microenvironments (25). Exosomes have been identified to transmit RNAs from cell to cell to affect biological influence. Herein, hinted by the published report that RNF157 mRNA was overexpressed in PCa tissues and exosomes, we analyzed the specific function of exosome-mediated RNF157 mRNA from PCa cells.

At first, we ensured that RNF157 mRNA expression was high in PCa tissues and cell lines. After co-culturing the PCa cells with macrophages, we noticed that such co-culture induced M2 polarization of macrophages in an exosome-dependent manner. To fathom out the regulatory mechanism, we carried out series of assays. Moreover, we found that co-culturing macrophages with PCa-cell-derived exosomes resulted in the up-regulation of RNF157 mRNA in M0 macrophages, which ultimately induce M2 polarization of macrophages.

Then, to probe into the downstream axis through which exosome-mediated RNF157 mRNA affected M2 polarization of macrophages, we searched for the target protein of RNF157 based on online prediction, as RNF157 is an E3 ubiquitin ligase (22). Ultimately, HDAC1 was chosen as our research target. Referring to previous studies, there are conflicts regarding to the functional role of HDAC1 in PCa. HDAC1 has once been recognized as a promoter of PCa by accelerating PCa cell proliferation (27). Current evidence has also claimed that HDAC1 could inhibit PCa cell invasion and PCa metastasis (28, 29). As to the role of HDAC1 regarding the polarization of macrophages, existing studies consistently show that the suppression of HDAC1 is able to facilitate M2 polarization (30–32). In this study, we found that RNF157 could facilitate the ubiquitination of HDAC1, thus destabilizing HDAC1 proteins. The destabilized HDAC1 ultimately resulted in the enhanced M2 polarization of macrophages. Moreover, our animal study revealed that RNF157 mRNA carried by exosomes could promote PCa tumor growth *via* promoting M2 polarization of macrophages.

However, this research did not further explore the downstream of exosomal RNF157 mRNA/HDAC1 axis in affecting the M2 polarization of macrophages in PCa. In former studies, it has been reported that HDAC1 coordinates with domain-associated protein 6 to inhibit the transcription of IL-6 (33). The suppressed HDAC1 has also been uncovered to induce higher STAT3 activity (29). Based on these findings, we conjectured that IL-6 and STAT3 might be the downstream of the exosomal RNF157 mRNA/HDAC1 axis in PCa, since both of them have been already proved to facilitate macrophage M2 polarization (34, 35). However, the exact downstream effector of RNF157/HDAC1 in PCa needs to be investigated in the future.

To conclude, this research work first unveiled that exosomal RNF157 mRNA secreted by PCa cells was able to induce M2 polarization of macrophages by destabilizing HDAC1 proteins, therefore accelerating PCa development. Our study provided a novel understanding concerning the molecular regulatory axis involving the participation of exosomes in PCa. More significantly, present work suggested that targeting RNF157 might be a promising method for PCa therapy.

## Data availability statement

The original contributions presented in the study are included in the article/**Supplementary Material**. Further inquiries can be directed to the corresponding authors.

## Ethics statement

The animal studies were approved by the Ethics Committee of the First Affiliated Hospital of Bengbu Medical College.

## Author contributions

HG, LM and JW administrated the whole research. SW and SY performed the experiments. HW and WS completed the figures. In the revised version, JW polished the article language and adjusted the layout of figures. The paper was co-written by ZC and MC. All authors contributed to the article and approved the submitted version.

## Funding

This work was supported by Youth Project of Natural Science Foundation of Anhui Province (2008085QH358); Excellent young teacher training object of ‘‘512 Talent Cultivation Program’’ of Bengbu Medical College (by51202306); Excellent Youth Science Fund of the First Affiliated Hospital of Bengbu Medical College (2019byyfyyq09).

## Acknowledgments

We are very grateful to all individuals and groups involved in this study.

## Conflict of interest

The authors declare that the research was conducted in the absence of any commercial or financial relationships that could be construed as a potential conflict of interest.

## Publisher’s note

All claims expressed in this article are solely those of the authors and do not necessarily represent those of their affiliated organizations, or those of the publisher, the editors and the reviewers. Any product that may be evaluated in this article, or claim that may be made by its manufacturer, is not guaranteed or endorsed by the publisher.

## Supplementary material

Supplementary Figure 1 M2 polarization of macrophages was promoted after co-culturing with PCa cells. **(A)** Light microscope was used to observe the change in cell morphology after PMA treatment and RT-qPCR evaluated the expression level of M0 maker (CD68). **(B)** Flow cytometry detected M2 polarization of macrophages before and after co-culturing with PC-3 cells that were treated with or without GW4869. **(C)** ARG1 and IL-10 expressions were analyzed *via* RT-qPCR in different groups. ^**^P < 0.01.

Supplementary Figure 2 Protein distribution was predicted on Hum-mPLoc 2.0.
