# Supplementary figures and images for: Exosomal RNF157 mRNA from prostate cancer cells contributes to M2 macrophage polarization through destabilizing HDAC1
Source: Front Oncol. 2022 Oct 3;12:1021270. doi: 10.3389/fonc.2022.1021270 (PMC9573993; doi:10.3389/fonc.2022.1021270)

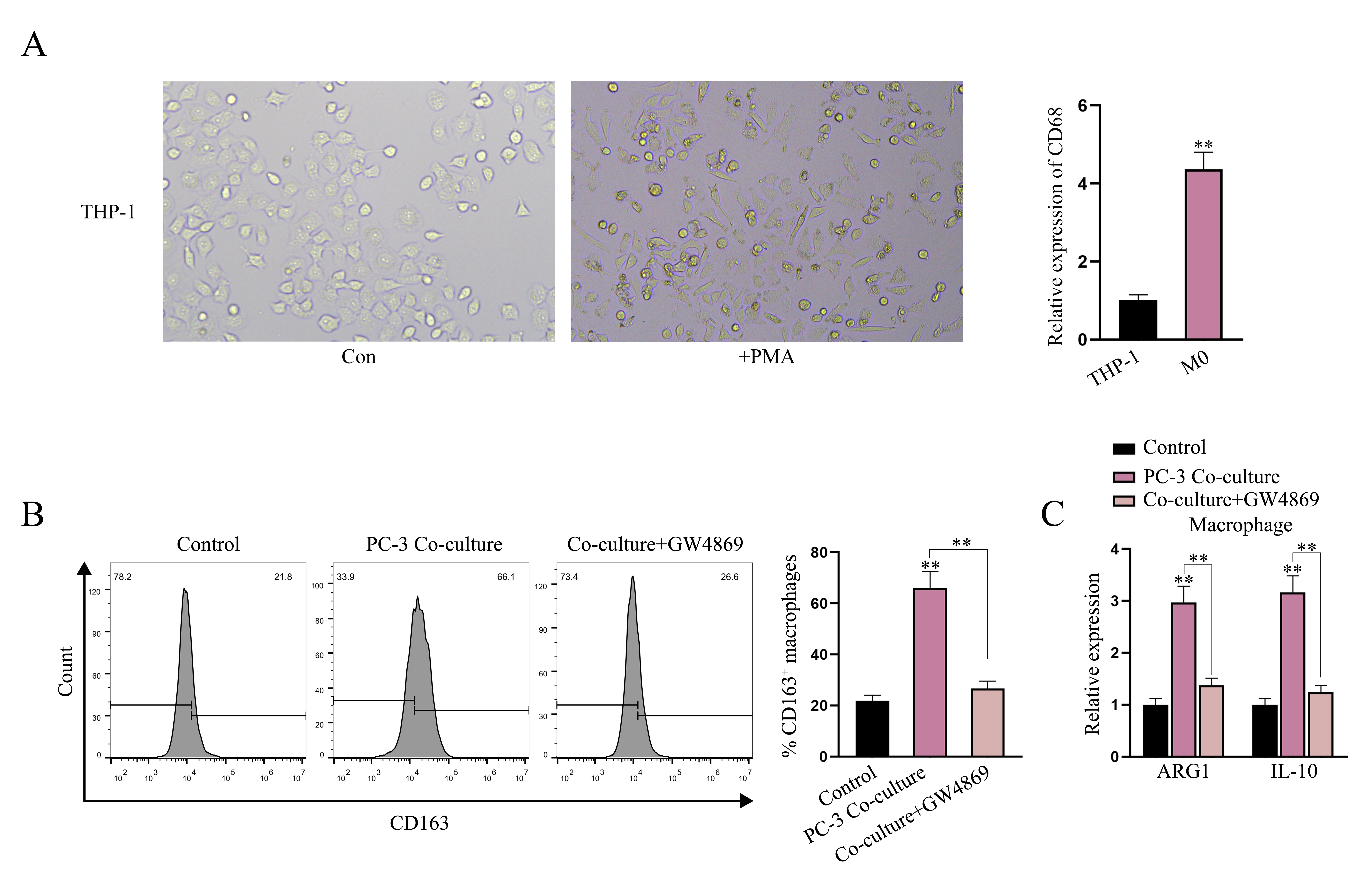

Supplement: Supplementary Figure 1 — M2 polarization of macrophages was promoted after co-culturing with PCa cells. (A) Light microscope was used to observe the change in cell morphology after PMA treatment and RT-qPCR evaluated the expression level of M0 maker (CD68). (B) Flow cytometry detected M2 polarization of macrophages before and after co-culturing with PC-3 cells that were treated with or without GW4869. (C) ARG1 and IL-10 expressions were analyzed via RT-qPCR in different groups. **P < 0.01. [file Image_1.tif]

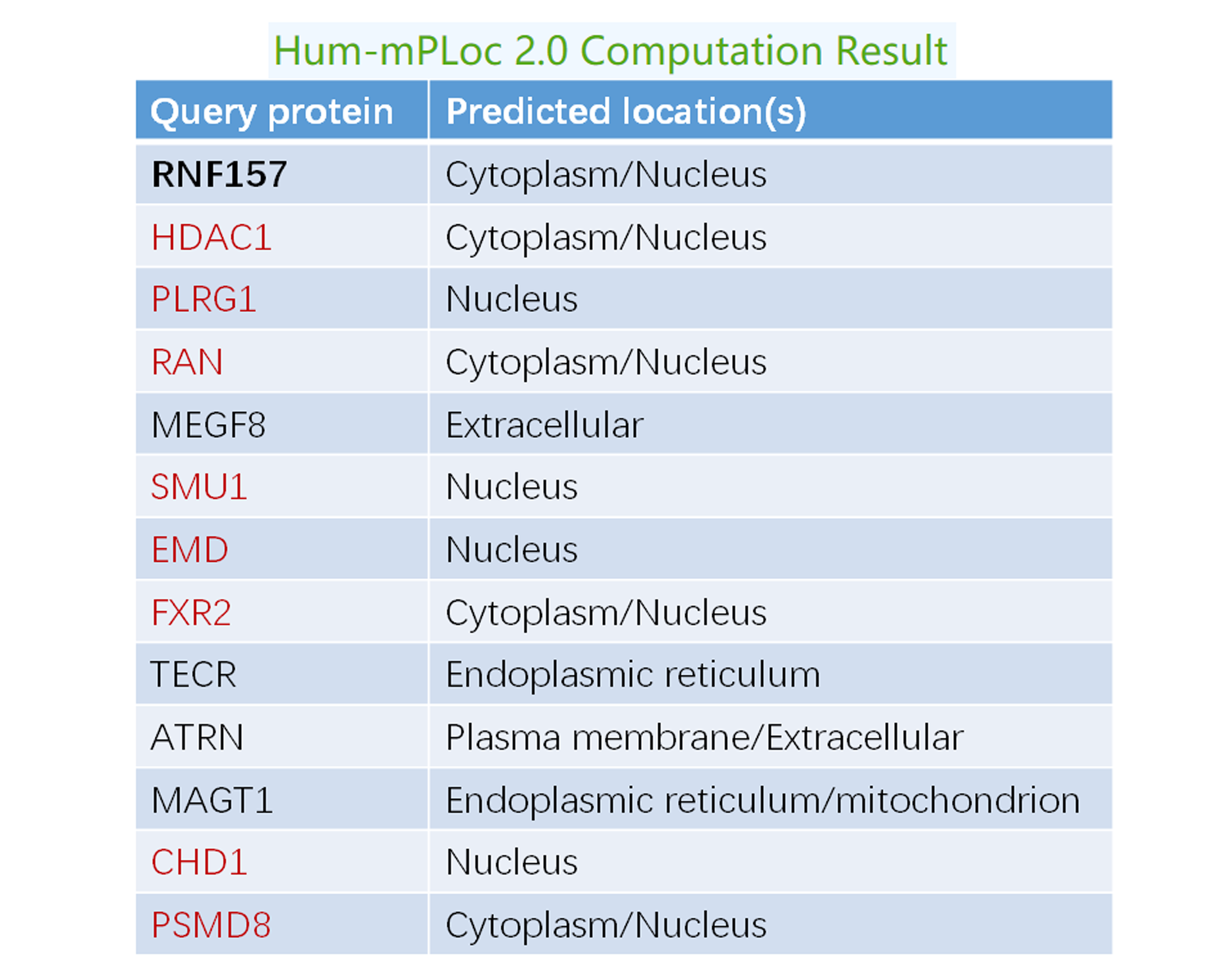

Supplement: Supplementary Figure 2 — Protein distribution was predicted on Hum-mPLoc 2.0. [file Image_2.tif]
